# Association between 9p21 genetic variants and mortality risk in a prospective cohort of patients with type 2 diabetes (ZODIAC-15)

**DOI:** 10.1186/1475-2840-11-138

**Published:** 2012-11-07

**Authors:** Gijs WD Landman, Jana V van Vliet-Ostaptchouk, Nanne Kleefstra, Kornelis JJ van Hateren, Iefke Drion, Klaas H Groenier, Rijk OB Gans, Harold Snieder, Marten H Hofker, Henk JG Bilo

**Affiliations:** 1 Diabetes Center, Isala Clinics, PO Box 10400, 8000 GK, Zwolle, The Netherlands; 2 Molecular Genetics, Department of Pathology and Medical Biology, University of Groningen, University Medical Center Groningen, Groningen, the Netherlands; 3 Department of Endocrinology, University of Groningen, University Medical Center Groningen, Groningen, the Netherlands; 4 Langerhans, Medical Research Group, Zwolle, the Netherlands; 5 Department of Internal Medicine, University Medical Center Groningen, Groningen, the Netherlands; 6 Department of General Practice, University of Groningen, University Medical Center Groningen, Groningen, the Netherlands; 7 Unit of Genetic Epidemiology and Bioinformatics, Department of Epidemiology, University of Groningen, University Medical Center Groningen, Groningen, the Netherlands; 8 Department of Internal Medicine, Isala Clinics, Zwolle, the Netherlands

**Keywords:** Type 2 Diabetes, CVD, Mortality, 9p21, Genetics, SNP

## Abstract

The genomic region at 9p21 chromosome near the *CDKN2A/CDKN2B* genes is associated with type 2 diabetes(T2D) and cardiovascular disease(CVD). The effect of the 9p21 locus on long-term mortality in patients with T2D has yet to be determined.

We examined three single nucleotide polymorphisms (SNPs) on 9p21, consistently and independently associated with T2D (rs10811661) or CVD (rs10757278, rs2383206), in relation to the risk of total and cardiovascular mortality in diabetic patients. We also aimed to replicate the previously observed interaction between rs2383206 and glycemic control on mortality.

Genotypes for three SNPs were determined in 914 individuals from a prospective cohort of T2D patients of Dutch origin. Associations with mortality were assessed using Cox proportional hazard analyses.

After a median follow-up of 9.5 years, 358 out of 914 patients had died. The hazard ratio (HR) for total mortality among individuals homozygous for the T2D-risk allele of rs10811661 compared to non-homozygous individuals was 0.74(95%CI 0.59-0.93). For the carriers of both CVD-risk alleles of rs10757278, the HR for total mortality was 1.31(95%CI 1.01-1.70). We found a significant interaction between rs2383206 and HbA1c on mortality, which was higher among patients with two CVD-risk alleles in the two lowest HbA1c tertiles (HR 1.68(95%CI 1.08-2.63); HR 1.48(95%CI 1.01-2.18).

In conclusion, common variants on 9p21 were associated with mortality in patients with T2D in a Dutch population. The T2D SNP was inversely associated with mortality, while the CVD SNP increased the risk for mortality. We confirmed a possible, although different, synergistic relationship between HbA1c and rs2383206 on total mortality.

## Introduction

Patients with type 2 diabetes (T2D) have a high risk for cardiovascular morbidity and mortality [1]. Multiple risk factors contribute to their development, including genetic factors [2-5]. Genome-wide association (GWA) studies for T2D [2-4] and cardiovascular disease (CVD) have identified the same susceptibility locus on chromosome 9p21 [5,6]. In this genomic region near the protein-coding genes *CDKN2A* and *CDKN2B* containing two adjacent linkage disequilibrium (LD) blocks the two single nucleotide polymorphisms (SNPs), rs10811661 and rs10757278, were consistently replicated as having an independent association with T2D and CVD, respectively [5,7,8].

Several studies indicated that the 9p21 locus might represent a genetic modulator in the cardiovascular system. Different pathophysiological pathways have been proposed, including the initiation and possibly progression of coronary atherosclerosis and development of multivessel coronary artery disease (CAD) [9-11]. One of these studies showed an effect of the 9p21 region on progression of plaques in the presence of already established CVD among non-diabetics, although this effect was different in patients with T2D [11]. In addition, a modulation of effect of common 9p21 variants on CVD risk by degree of glycemic control has been reported [12]. Doria et al. reported an increased risk of CAD and mortality in the presence of poor glycemic control in T2D patients and also suggested a synergism between the high ‘CVD’-risk genotype of rs2383206 and poor glycemic control on mortality [12]. Whether the polymorphisms on 9p21 can predict overall or cardiovascular mortality in patients with T2D and how this association is modulated by glycemic control is still to be determined.

In this study we aimed to investigate the total and cardiovascular mortality risk in association with the ‘T2D‘SNP rs10811661 and the ‘CVD’ SNP rs10757278 in a prospective cohort study of type 2 diabetes patients of Dutch origin. Next, we aimed to replicate the findings obtained by Doria et al. on the interaction between the rs2383206 variant and level of glycemic control and its effects on total and CVD mortality.

## Methods

### Study population

In 1998, a large shared-care diabetes project was initiated: the Zwolle Outpatient Diabetes project Integrating Available Care (ZODIAC) [10]. Of all the patients that were invited, 90% participated in this study and 10% were excluded or refused to participate. Type 2 diabetes was defined according to the World Health Organization (WHO) criteria (random plasma glucose level >11.1 mmol/l or a fasting plasma glucose level >7.0 mmol/l) [13]. Patients with a very short life expectancy, with insufficient cognitive abilities and those treated by an internist were excluded. At the end of the project’s first year (1998) and the start of the second year (1999), blood was taken and stored for future research and quality control. The majority of the participants were of Dutch Caucasian origin [11]. Baseline data involved recording a full medical history including the presence of any macrovascular complications, medication use and tobacco exposure. Patients were considered having macrovascular complications if they had a history of angina pectoris, myocardial infarction, percutaneous transluminal coronary angioplasty, coronary artery bypass grafting, stroke or transient ischemic attack. Laboratory and physical assessment data were collected and included glycated hemoglobin (HbA1c), nonfasting lipid profile, serum creatinine (SCr, a kinetic colorimetric Jaffé method [Modular P Analyzer; Roche, Almere, the Netherlands] was used), albumin-to-creatinine ratio (ACR) using immunonephelometry (Behring Nephelometer; Behringwerke, Mannheim, Germany), blood pressure (measured twice with a Welch Allyn sphygmomanometer in the supine position after at least 5 min of rest). Height and weight were measured while the participants were dressed in light clothing and without shoes. HbA1c was measured by affinity-based high-performance liquid chromatography on a primus CLC 385 (Primus Diagnostics, Kansas city, Missouri, USA) by the Isala Clinics Laboratory.

Life status and cause of death were retrieved in 2009 from records maintained by the hospital and the GPs. Causes of death were coded according to The International Classification of Diseases, 9th Revision (ICD-9). DNA samples were available for 914 T2D patients. The ZODIAC study and the informed consent procedure were approved by the local medical ethics committee. Informed consent was obtained from all patients.

### Genotyping

**Figure 1:**
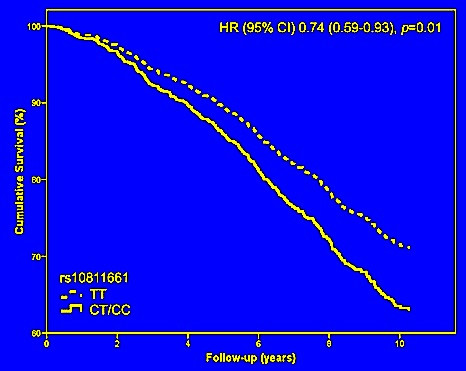
**Pairwise linkage disequilibrium** (**LD) plot, calculated as r**^**2**^**, among genotyped SNPs.** Red and white colours indicate high and low level of LD, respectively. The r^2^ measures of LD are shown in the squares.

Based on the original GWA scan by Helgadottir et al. [8], we selected the SNPs rs10811661 and rs10757278 on the 9p21 locus reported to be strongly associated and consistently replicated with T2D and CVD, respectively, in European populations [5,6,8]. These variants and the SNP rs2383206 (LD with rs10757278 r^2^=0.86 (HapMap phase III, release 2)) from the study by Doria et al. [12] were genotyped using TaqMan assays (Applied Biosystems, Nieuwekerk a/d IJssel, The Netherlands). Assays were performed according to the manufacturer’s specifications and the genotypes were analyzed using a TaqMan 7900HT (Applied Biosystems). The DNA samples were processed in 384-well plates and each plate contained 16 genotyping controls (4 duplicates of 4 different Centre d’Etude du Polymorphisme Humain (CEPH) samples). There were no discordances in the genotypes of any of the CEPH samples or the CEU data available from HapMap. The genotype success rates were 97.8%, 96.6% and 98.5% for rs10811661, rs10757278 and rs2383206, respectively. Figure 1 shows the LD plot among the three genotyped SNPs of our study.

### Statistical analysis

Univariate analysis was performed to test the relationships between the genotypes and baseline variables (i.e. age, gender, diabetes duration, history of macrovascular complications, smoking status, body mass index (BMI), Hba1c (%), systolic blood pressure, serum creatinine, total cholesterol-HDL ratio, and urinary albumin-to-creatinine ratio) using a student’s *t*-test for normally distributed data and a Mann Whitney *U* test for non-normally distributed data. Associations of the SNPs with macrovascular disease and other metabolic factors at baseline were assessed with univariate and by multivariate logistic regression analysis. In this model, adjustment was made for 11 variables.

A Cox proportional hazard model was constructed to assess the association between the SNPs rs10811661 and 10757278 and the primary endpoints of total and cardiovascular mortality after follow-up. SNPs were coded by genotype as 0,1, and 2 with individuals homozygous for the non-risk allele as the reference group. The risk and non-risk alleles were defined on the basis of the results from the discovery GWAS studies [2,4,6]. The recessive genetic model was selected prior to the analysis in conjunction with all previous prospective studies [12,14-17], i.e. the carriers of two copies of the risk allele (genotype 2) were compared versus the heterozygotes or non-carriers of the risk allele (genotypes 1 or 0).

We used two different models. In model 1, adjustment for age and gender was included, while model 2 included nine additional baseline variables as mentioned above and also in Table 1. All variables, except gender, smoking status and macrovascular complications were added as continuous variables.

**Table 1:** Baseline characteristics of the study population

**Characteristics**	**Total number of patients**
	N=914
Age (in years)	67.9 (±11.3)
Sex (% women)	57.8
Diabetes duration (in years)	5.0 (2–10)
Smoking (%)	18.1
Macrovascular complications (%)	31.1
BMI (kg/m^2^)	28.9 (±4.6)
HbA1c (%)	7.3 (±1.1)
Systolic blood pressure (mmHg)	152.5 (±25.1)
eGFR (ml/min/1,73 m^2^)	74.3 (±27.1)
Total cholesterol-HDL ratio	5.0 (±1.4)
Albumin creatinine ratio	1.9 (1.0-5.9)

Data are means ±SD or median with interquartile range for non-normally distributed data or %.

For the replication analysis of the rs2383206 by HbA1c interaction, we constructed a Cox proportional hazard model with the primary endpoint of total and cardiovascular mortality. In conjunction with the study by Doria [12], the presence of interaction was tested by adding a cross-product term to the Cox proportional hazard model including glycemic control and rs2383206 genotype using the same genetic model as in study by Doria et al., i.e. the recessive model [12], together with age at baseline and sex as main effects. If interaction could be confirmed, we then repeated the multivariate analysis stratified according to the HbA1c subgroups. In concurrence with the aforementioned study, HbA1c was categorized in tertiles [12].

For cardiovascular mortality, all competing deaths were treated as censored observations at time of death. Patients without a known cause of death were censored in this analysis. Continuous variables were centered to avoid co-linearity. The proportional hazards assumption was examined using log (−log) survival plots. The proportional hazard assumptions were met for all analyses. All analyses were performed with SPSS version 15.0.1 (SPSS inc., Chicago, Illinois, USA), and a two-sided p-value of <0.05 was considered statistically significant.

## Results

The baseline characteristics of the study population are presented in Table 1. The average age was 68 years with a median diabetes duration of 5 years, 31% had macrovascular complications at baseline and the average HbA1c was 7.3%. During a median follow-up period of 9.5 years, 358 (39%) patients had died. Cause of death was known for 336 (94%) patients. The proportion of deaths attributable to cardiovascular diseases was 41% (n=146).

In the univariate analysis, all the baseline characteristics - age, gender, duration of diabetes, smoking status, macrovascular complications at baseline, BMI, HbA1c, systolic blood pressure, serum creatinine, total cholesterol to HDL-cholesterol ratio, and albuminuria creatinine ratio – were not significantly different between the groups according to the rs10811661, rs10757278 or rs2383206 genotypes. Also, insulin use was not different according to the different genotypes.

In the multivariate analysis, patients with the ‘CVD’-risk genotype of rs107577278 had an increased risk for macrovascular complications at baseline compared to the non-carriers (OR 1.65 (95% CI 1.08-2.36, *p*=0.02)). There were no associations between the genotypes for rs10811661 and rs2383206 and macrovascular disease at baseline. All other baseline variables in the cross-sectional analysis were not associated with the T2D and CVD risk alleles.

### The ‘T2D’ risk variant rs10811661 and mortality

**Figure 2:**
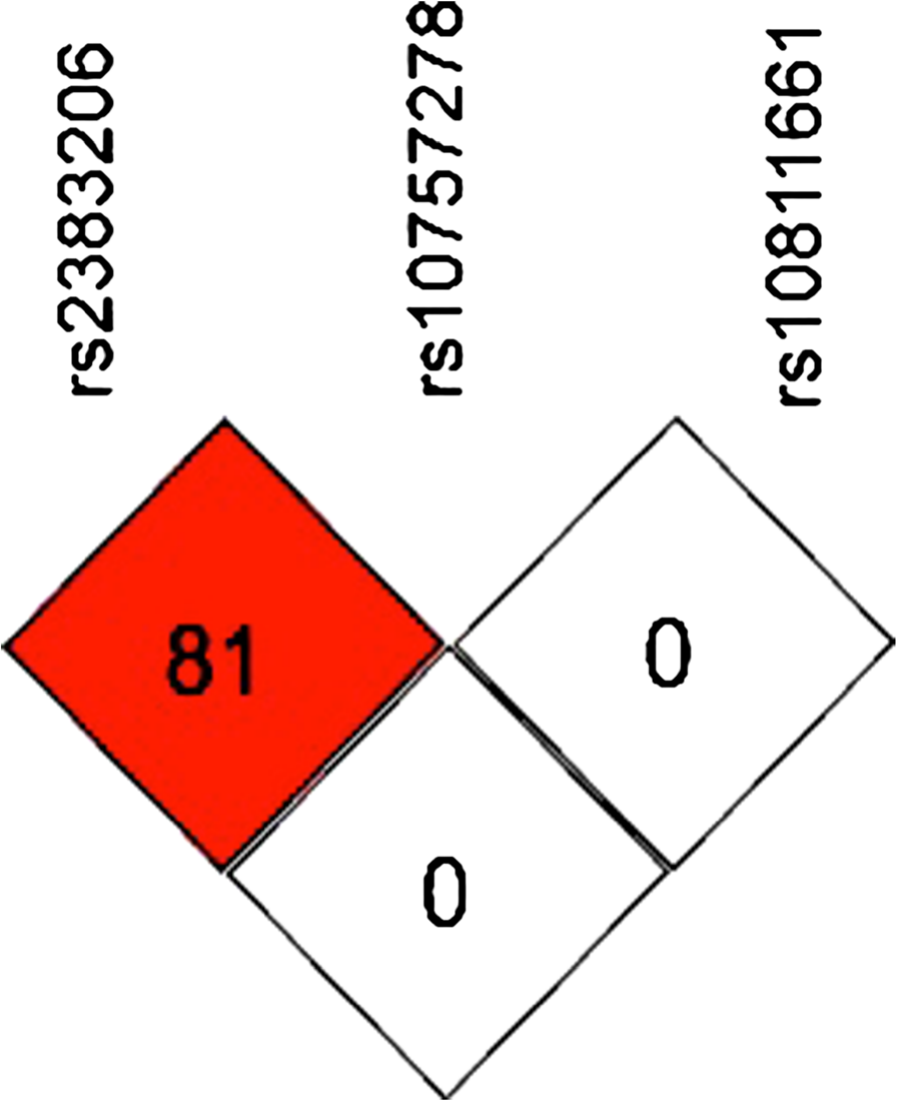
**Total mortality in type 2 diabetes patients in relation to the rs10811661 genotypes.** Survival curves indicate a lower risk for total mortality in carriers of both risk alleles of the ‘T2D’ variant rs10811661 compared to patients either homozygous or heterozygote for the non-risk allele. The HR was adjusted for multiple baseline covariates (model 2).

In our study, the hazard ratio (HR) for total mortality in the patients carrying both ‘T2D’- risk alleles (the TT- genotype) was 0.79 (95%CI 0.63-0.99, *p*=0.04) for model 1 and 0.74 (95%CI 0.59-0.93, *p*=0.01) for model 2 compared to carriers of the non-risk allele (the CT and CC genotypes), see Table 2 and Figure 2. Although the effect size was similar with a HR of 0.76, there was no significant relationship between rs10811661 and cardiovascular mortality (Table 2).

**Table 2:** Cox proportional hazard analyses for total and cardiovascular mortality among type 2 diabetes patients according to the rs10811661 and rs10757278 genotypes after a median follow-up of 9.5 years

**Genotype**	**N of patients (deceased)**	**HR total mortality**^**a**^	**HR total mortality**^**b**^	**HR CV mortality**^**a**^	**HR CV mortality**^**b**^
rs10811661					
CT/CC	264 (113)	1	1	1	1
TT	635 (238)	0.79 (0.63 -0.99)	0.74 (0.59-0.93)	0.82 (0.57-1.17)	0.76 (0.52-1.10)
Missing	15 (7)	*p=*0.04	*p=*0.01	*P*=0.28	*p*=0.15
rs10757278					
AA/AG	704 (266)	1	1		1
GG	188 (81)	1.33 (1.04-1.71)	1.31 (1.01-1.70)	1.27 (0.87-1.72)	1.18 (0.80-1.78)
Missing	22 (11)	*p* =0.02	*p* =0.04	*P*=0.20	*p*=0.44

^a^ Corrected for age and sex according to model 1, ^b^ corrected for 11 baseline variables (age, gender, diabetes duration, history of macrovascular complications, smoking status, BMI, Hba1c, systolic blood pressure, serum creatinine, total cholesterol-HDL ratio, and urinary albumin-to-creatinine ratio) according to model 2.

### The ‘CVD’ risk variant rs10757278 and mortality

The risk of total mortality was higher in individuals carrying both risk alleles of rs10757278 (the GG-genotype) as compared with patients heterozygous or homozygous for the non-risk allele (the AA and AG genotypes). For model 1 the HR was 1.33 (95%CI 1.04-1.71, *p*=0.02) (Table 2). For the fully adjusted model 2 the HR was 1.31 (95%CI 1.01-1.70, *p*=0.03). We found no significant association between rs10757278 and cardiovascular mortality (Table 2).

### Replication of the interaction between rs2383206 and glycemic control on mortality

Patients with two copies of the risk allele of rs2383206 (the GG-genotype) as compared to patients carrying the non-risk allele (the AA and AG genotypes) had a significantly greater risk of dying. The age and sex adjusted HR was 1.31 (95%CI 1.03-1.65, *p*=0.03) for total mortality and 1.30 (95%CI 0.90-1.88, *p*=0.17) for cardiovascular mortality. A significant interaction between rs2383206 and glycemic control in the analysis of total mortality was detected in the Cox proportional hazard analyses (for the genotypes: GG versus AA/AG, *p*=0.02). There was no significant interaction between rs2383206 and glycemic control in the analysis with cardiovascular mortality (*p*=0.17) and therefore we only investigated the effect of the SNP with total mortality stratified according to HbA1c tertiles. The age and sex adjusted relationship between the rs2383206 genotypes and total mortality was only significant in the lower two HbA1c tertiles (HbA1c ≤6.7% and HbA1c 6.7% to 7.7%) (Table 3). For model 1 the HR was 1.68 (95% CI 1.08-2.62, *p*=0.02), 1.48 (95%CI 1.01-2.19, *p*=0.04) and 0.92 (95% CI 0.60-1.41, *p*=0.71) for the HbA1c categories HbA1c ≤6.7%, HbA1c 6.7%-7.7% and >7.7%, respectively.

**Table 3:** Cox proportional hazard analysis for total mortality by degree of glycemic control among type 2 diabetes patients according to the SNP rs2383206 genotype after a median follow-up of 9.5 years

**Genotype**	**N of patients (deceased)**	**Hazard ratio total mortality**^**a**^		
		**HbA1c 1st tertile (≤6.7%)**	**HbA1c 2nd tertile (6.7%-7.7%)**	**HbA1c 3rd tertile (≥7.7%)**
rs2383206				
AA/AG	674 (252)	1	1	1
GG	222 (96)	1.68 (1.08-2.62)	1.49 (1.01-2.19)	0.92 (0.60-1.41)
Missing	18 (10)	*p*=0.02	*p*=0.04	*p*=0.71

^a^ Corrected for age and sex.

## Discussion

This prospective study shows that three genetic risk variants for T2D and CVD on 9p21 are associated with total mortality in patients with T2D. We found that being homozygous for the diabetes risk allele rs10811661 was protective for total mortality. We confirmed the previously reported association between two cardiovascular risk alleles of the rs10757278 and rs2383206 variants with an increased mortality risk. We also confirmed a possible synergistic effect of glycemic control with the ‘CVD’ variant rs2383206 on mortality. However, this effect was present in the patients with good glycemic control, not in individuals with a poor glycemic control as was shown previously [12].

All three SNPs rs10811661, rs10757278 and rs2383206 are located in adjacent LD blocks of the 9p21 locus and represent independent genetic markers for susceptibility to T2D (rs10811661) and CVD (rs10757278 and rs2383206, in high LD with each other (r^2^=0.86)) [16]. The closest genes in proximity of this region are *CDKN2A* and *CDKN2B,* both of which are highly expressed in pancreatic islet cells; *CDKN2A* plays a role in pancreatic islet regenerative capacity [2]. To date, no evidence of an association between the ‘T2D‘variant, rs10811661, and *CDKN2A* and *CDKN2B* gene expression in three relevant human tissues (i.e. the colon, liver and pancreas) was found [18]. In contrast, the rs10757278 from the ‘CVD’ risk interval on 9p21 has been shown to regulate cardiac *CDKN2A/B* expression [19]. Recently, Harismendy et al. reported that the 9p21 locus is enriched in enhancers including one that interacts with the risk allele of rs10757278, emphasizing the important role of rs10757278 [20]. The risk allele disrupts a binding site for STAT1, the signal transducer for interferon-γ and cytokines. And thus suggesting a direct mechanistic link between the 9p21 region and atherosclerosis risk via alteration of vascular cell proliferation [19-21].

### Association of the ‘T2D’9p21 variant with mortality

We found that having two ‘T2D‘risk T-alleles of the rs10811661 variant was associated with a decreased total mortality risk. One possible explanation of the observed relationship between the genetic risk to T2D and lower mortality could be that subjects who are genetically prone to develop diabetes could develop this disease in the relative absence of lifestyle related diabetes risk factors. These lifestyle related factors, for example, increased general and central adiposity are also associated with chronic diseases and mortality [22,23]. Although our study included only a limited number of patients with newly diagnosed diabetes, there were no significant differences in baseline variables, for example in BMI, between patient’s homozygous vs. non-homozygous for the T2D risk allele.

Another explanation could be that the observed relationship between the ‘T2D’ risk allele of rs10811661 and low mortality in our study was in fact caused by its protective effect on other diseases. Interestingly, Beekman et al. have recently found a positive relationship, although non-significant, between the two ‘T2D’ risk alleles of rs10811661 and longevity (OR 1.11 (95%CI 0.96-1.23)) [24], which is in agreement with our results. This hypothesis may also be supported by recent studies reporting an association between increased T2D genetic susceptibility and reduced prostate cancer risk [25,26]. In our study, we found no significant relationship with the ‘T2D’-risk allele and cancer mortality, although our sample size was probably too small to detect this association.

Finally, it is important to note that no underlying causative mechanisms linking the genetic marker rs10811661 with the pathophysiology of T2D were revealed yet. Further studies are needed to confirm this relationship and should also focus on the discovery of potential molecular and physiological pathways explaining T2D development.

### Interaction between the ‘CVD’9p21 variant and glycemic control on mortality

We observed an association between the homozygote CVD-risk genotypes for rs10757278 and rs2383206 and an increased risk of total mortality. Although there was an increased risk for macrovascular complications at baseline in patients with the ‘CVD’-risk genotype of rs10757278, the association with the 9p21 variant and cardiovascular mortality after follow-up was not significant. The most likely explanation is that our study had a lack of power to detect a difference in cardiovascular mortality. Another explanation could be that in the patients with T2D, the CVD-risk genotype influences the initiation of atherosclerosis but not progression of atherosclerosis [11].

In our study, the effect of the rs2383206 variant on total mortality in diabetic patients was further influenced by the degree of glycemic control. The effect of the risk allele disappeared in individuals within the highest HbA1c tertile. These results possibly suggest that for the patients with low levels of HbA1c compared to the patients with poor glycemic control, total mortality is determined to a larger extent by genetic factors. Indeed, in a previous ZODIAC study, we showed that HbA1c is an important factor with regard to mortality risk, but only in the patients with higher HbA1c level [27]. In contrast with this, Doria et al. reported that cardiovascular and total mortality associated with the 9p21 variant rs2383206 were higher in patients with poor glycemic control [12]. Although we used the same statistical model as Doria et al. [12], we could not confirm the synergism between rs2383206 and poor glycemic control on mortality, neither did we observe an interaction between HbA1c level and rs2383206 with cardiovascular mortality. The difference in these two observations can be explained by various factors. First, HbA1c at baseline was substantially lower in our cohort compared to the HbA1c level in the prospective sample reported by Doria et al. (7.3±1.1 versus 8.3±1.4, respectively) [12]. Secondly, the study by Doria et al. used multiple HbA1c measurements before the study recruitment. Unfortunately, these data were not available in the ZODIAC study. Finally, it is possible that the strength of the association reported by Doria et al. may have been overestimated due to selective forwarding of especially severe CAD cases to the medical centers from which participants were recruited in the above mentioned study. Thus, it remains unclear to what extent differences between the study populations, for example, in the baseline HbA1c level or in the inclusion criteria, influenced the relationships between glycemic control and mortality in the carriers of two risk alleles of rs2383206. Hence, additional replication studies are necessary to further investigate the interaction between the 9p21 locus and glycemic control.

### Strengths and limitations

The unique strengths of our study include its prospective study design using a cohort of T2D patients with a long follow-up for incident mortality, and the availability of the baseline covariates to adjust for in the survival analysis. Also, we selected three 9p21 genetic variants based on the original GWA scans and the replication studies in Europeans [5,6,8,12]. Recently a comprehensive fine-mapping of the 9p21 region using the targeted sequencing and the reference panel from 1000 Genome project has been performed [28]. Although an additional set of common associated variants was identified, no stronger associations than the original GWAS signals have been revealed.

It needs to be noted that the a priori selection process of the SNPs could be the subject of debate. For example, other ‘CVD’ associated variants in the 9p21 region such as rs1333040 or 1333049 were also reported as independently associated with cardiovascular disease [29]. However, rs1333040 and 1333049 are in high LD with rs10757878 (r^2^=0.57 [6] and r^2^=0.93 [29] for rs1333040 and 1333049, respectively). Moreover, it has recently been shown that the CVD risk allele of rs10757278 is located in one of the enhancers and disrupts a binding site for the transcription factor STAT1, thus, highlighting the potential functional importance of rs10757278 [20]. Another limitation of our study is the number of events that may have been insufficient to draw valid conclusions on cause specific mortality, i.e. cardiovascular mortality. A third potential limitation concerns that only the recessive genetic model was tested in our analysis. To alleviate the multiple testing problems in the data by reducing the number of statistical tests we have exclusively performed our analysis according to the recessive model as a predefined model. This model was selected a priori and based on the reports from all previous prospective studies [12,14,15,17], although we acknowledge that some previous cross-sectional studies have also used the additive model in their analyses [5].

## Conclusion

In conclusion, our study provides evidence for associations between genetic variants on 9p21 with mortality in T2D patients. We found that the genetic risk variant for T2D was inversely associated with mortality, while the risk variant for CVD increased the risk for mortality. We also found that the effect of the ‘CVD’-risk variant on mortality was further influenced by the degree of glycemic control. Altogether, these observations indicate heterogeneity in the association patterns between the 9p21 common variants and mortality and highlight the central role of this genomic region on disease outcome in T2D patients. Future studies should focus on investigating the relationship between genetic and non-genetic risk factors in the complex aetiology of T2D and its complications.

## Abbreviations

T2D: Type 2 diabetes; CI: Confidence interval; CEPH: Centre d’Etude du Polymorphisme Humain; CVD: Cardiovascular disease; HR: Hazard ratio; HWE: Hardy Weinberg equilibrium; LD: Linkage disequilibrium; OR: Odds ratio; SD: Standard deviation; SNP: Single nucleotide polymorphism; GWA: Genome-wide association.

## Competing interests

The authors state that they have no conflicts of interest.

## Authors’ contributions

GWDL: researched data, contributed to discussion, drafted manuscript. JVvVO: carried out the molecular genetic studies, participated in the sequence alignment, contributed to discussion and the drafting of the manuscript. NK, KJJvH, ID, ROBG, CW, HS, MHH and HJGB: contributed to discussion, reviewed manuscript. KHG: researched data/ statistician, reviewed manuscript. All authors read and approved the final manuscript. Both GWDL and JVvVO contributed equally to this paper.
